# Clinical features and treatment response to differentiate idiopathic peritonitis from non-strangulating intestinal infarction of the pelvic flexure associated with *Strongylus vulgaris* infection in the horse

**DOI:** 10.1186/s12917-022-03248-x

**Published:** 2022-04-23

**Authors:** Ylva Hedberg-Alm, Eva Tydén, Lena-Mari Tamminen, Lisa Lindström, Karin Anlén, Maria Svensson, Miia Riihimäki

**Affiliations:** 1 Department of Biomedical Science and Veterinary Public Health, Parasitology Unit, Swedish University of Agricultural Sciences, Uppsala, Sweden; 2 Equine Clinic, University Animal Hospital, Swedish University of Agricultural Sciences, Uppsala, Sweden; 3 Department of Clinical Sciences, Veterinary Epidemiology Unit, Swedish University of Agricultural Sciences, Uppsala, Sweden; 4 Department of Biomedical Science and Veterinary Public Health, Pathology Unit, Swedish University of Agricultural Sciences, Uppsala, Sweden; 5 Specialist Equine Hospital, Evidensia Animal Hospital, Helsingborg, Sweden; 6 Department of Clinical Sciences, Equine Medicine Unit, Swedish University of Agricultural Sciences, Uppsala, Sweden

**Keywords:** Equine, Intestinal parasite, Colic, Rectal examination, Prognosis

## Abstract

**Background:**

Peritonitis in horses secondary to non-strangulating infarction (NSII) has a guarded prognosis, even after intestinal resection. In contrast, horses with idiopathic peritonitis respond well to medical treatment. Affected horses in both cases often show signs of both colic and systemic inflammation, but early diagnosis is crucial for optimal treatment and an accurate prognosis. One cause of NSII is thrombus formation secondary to *Strongylus vulgaris* larval migration. There has been a documented increase in *S. vulgaris* prevalence in Sweden since the implementation of selective anthelmintic treatment in 2007, which subsequently could result in a rise in NSII cases. In a retrospective clinical study, medical records from cases diagnosed with NSII of the pelvic flexure or idiopathic peritonitis from three equine referral hospitals in Sweden during 2017–2020 were reviewed. Information including demographic data, relevant medical history, and clinical- and laboratory parameters were obtained from patient records. To facilitate the differentiation between cases of idiopathic peritonitis and cases with confirmed NSII of the pelvic flexure, the aim of the study was to compare clinical and laboratory parameters, clinical progression and initial response to antimicrobial treatment. A secondary aim was to compare survival-rates.

**Results:**

Horses with NSII (*n* = 20) were significantly more likely to present during the winter months with a poorer response to medical treatment within 48 h. Cases of idiopathic peritonitis (*n* = 107) had a 100% survival rate with medical treatment, although one case required surgical correction of a colon displacement. In comparison, all confirmed NSII cases were non-responsive to antimicrobial treatment, with a survival rate to discharge of 50% after colon resection. Specific rectal findings and peripheral blood neutropenia were strongly associated with NSII.

**Conclusions:**

In Sweden, idiopathic peritonitis cases still predominate over *S. vulgaris* associated NSII cases and have an excellent survival rate with antimicrobial treatment. However, horses presenting with septic peritonitis during the winter months with a palpable rectal mass and displaying fever and colic signs beyond 48 h of medical treatment are likely to suffer from NSII of the pelvic flexure and should be considered for abdominal surgery.

**Supplementary Information:**

The online version contains supplementary material available at 10.1186/s12917-022-03248-x.

## Background

Acute primary or idiopathic peritonitis without an identifiable aetiology is reported to have very high survival rates (94–100%) and although often described as uncommon, the disease is well recognized in some countries, such as Australia and Sweden [1–3]. Peritonitis secondary to non- strangulating intestinal infarction (NSII), however, is associated with a grave prognosis with medical treatment alone and poor prognosis even after surgical resection of the affected intestinal segment (33%) [4]. Although several other causes of NSII have been suggested, [5–7] infarctions of the intestinal wall secondary to mesenteric arterial thrombosis associated with *Strongylus vulgaris* larval migration have been well described [4, 8, 9]. Since the implementation of prescription-only anthelmintic treatment, and a more selective anthelmintic treatment strategy, Sweden and Denmark have documented an increase in *S. vulgaris* prevalence in the equine population [10, 11]. Recently, ELISA antibodies to *S. vulgaris* were shown to be present in 62% of horses, revealing a high level of exposure to the parasite in the Swedish horse population [12].

Clinical cases of both NSII and idiopathic peritonitis often display varying degrees of colic accompanied by signs of systemic inflammation [3, 4]. In addition, both diseases present without signs of trauma or a history of potential risk factors, such as recent parturition or abdominal surgery. A study performed in Denmark suggested that, in areas where *S. vulgaris* is endemic, all horses admitted with septic peritonitis of an unidentifiable cause should undergo exploratory laparotomy [4]. However, since idiopathic peritonitis is commonly identified in Swedish horses [3], distinguishing between the two disease forms is important, both for optimal treatment and an accurate prognosis.

To enable early differentiation between cases of idiopathic peritonitis and cases with confirmed NSII, the main aim of the present study was to compare clinical and laboratory parameters, clinical progression and initial response to antimicrobial treatment between horses diagnosed with idiopathic peritonitis and horses with confirmed NSII of the pelvic flexure, using medical records from three Swedish equine referral hospitals during a four year period (2017–2020). A secondary study aim was to compare the survival rates to discharge between these two case groups.

## Results

### Included cases

A total of 127 cases were included in the study and further divided into two groups: i) 107 cases were classified as idiopathic peritonitis without an identifiable cause of the peritonitis as evaluated by clinical and rectal examination ii) 20 cases had confirmed NSII of the pelvic flexure, determined by abdominal surgery and pathological assessment of the resected intestine or full body necropsy.

### Excluded cases

Fifteen cases were excluded from the main analyses, as they did not meet the set inclusion criteria for case selection. Of these, two cases had suspected parasitic lesions involving the jejunum. One case was suspected of NSII at the pelvic flexure, but the resected intestine was not sent for pathological assessment, and one case had a small infarction in the colon that was not resected. The remaining 11 cases had rectal examinations, in some cases including rectal ultrasonographic findings, suggestive of a mass, thickened intestinal wall at the pelvic flexure, adhesions and/or suspected thrombus formation in the mesenteric vessels, without confirmative diagnosis, either due to medical treatment response (*n* = 8) or lack of necropsy (*n* = 3). Although unconfirmed, it was acknowledged that some of these latter horses could represent NSII cases that responded to medical treatment and the progression of clinical signs and outcome in this group was therefore recorded. In addition, this group of unconfirmed cases was included in the cluster analysis.

### Exploratory and descriptive analyses using cluster analysis

For an initial descriptive analysis of the available data, a cluster analysis including demographic-, clinical- and laboratory parameters as well as outcome in idiopathic peritonitis cases (*n* = 107), confirmed non-intestinal infarction (NSII) cases (*n* = 20) and unconfirmed NSII cases (*n* = 11) was employed to reveal associations between individual horses in the dataset (Suppl. Figure 1). Clinical diagnosis clustered closely to the parameters leucocyte count, neutrophil count, specific rectal findings (rectal mass/adhesions or thickened intestinal wall) as well as the outcome parameters euthanized and days at clinic (see box “a”, Suppl. Figure 1). The pattern suggested that confirmed NSII was associated with lower leucocyte and neutrophil counts. The majority of individuals with confirmed NSII showed high similarity and clustered together (see box “b”, Suppl. Figure 1). The common aspects of the individuals in cluster “b” were that they presented primarily during the winter months with a rectal mass/adhesion or a thickened intestinal wall and a poor outcome.

### Demographic data

In the idiopathic peritonitis group, Warm-blooded horses were most common ( 50%), followed by pony breeds ( 18%), Icelandic horses (17%), Standardbred trotters (6%), Cold bloods (5%) and Thoroughbreds (5%). One horse was of unknown breed. Similarly, Warmbloods predominated in the NSII group ( 67%), with 17% Cold bloods, one Icelandic horse, one of pony breed and one Arabian. In two cases in the NSII group the breed was not recorded. The idiopathic case group consisted of 49% geldings, 44% mares and 7% stallions. Of the NSII confirmed cases, 65% were geldings, and 30% mares, with one stallion. The mean (± SD) and median age in the two groups were as follows: idiopathic 12.7 (± 6.0) and 12 years; NSII 11.7 (± 4.8) and 10.5 years. There were no significant differences in demographic data between groups (Suppl. Table 1a).

### Previous medical history and admission data

**Fig. 1:**
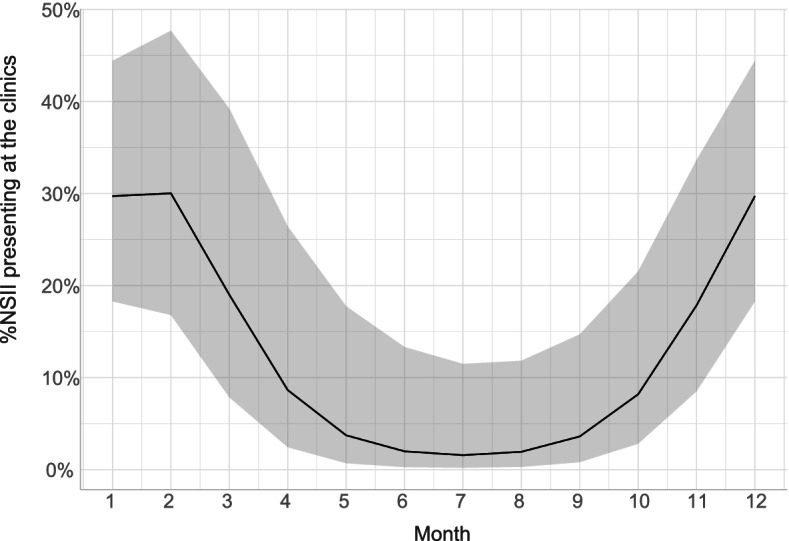
Predicted probabilities of a confirmed non-strangulating intestinal infarction (NSII) diagnosis according to month of presentation. The x-axis shows month of prestentation, where the numbers 1–12 represents the months of January through December. The y-axis shows the proportion (presented as a percentage) of admitted peritonitis cases with a confirmed NSII diagnosis

No statistical differences in relevant medical history data were found (Suppl. Table 1b). Within both groups, the number of cases that had received anthelmintic treatment within six months of presentation was similar to the number not treated within this time-frame, although this data was missing in a large number of cases in the idiopathic case group ( 39%). In total, seven cases with confirmed NSII lesions had been treated with an anthelmintic drug within six months of admission. Variables with a significant difference between the two study groups are presented in Table 1. The univariable analysis (as well as the cluster analysis) suggested that horses with confirmed NSII were more likely to present during the winter months (OR = 10, Table 1; Suppl. Figure 1). The generalized additive model confirmed a significant cyclical trend (*p* < 0.001) where horses with confirmed NSII were more likely to present during the winter months (Fig. 1). Horses later confirmed with NSII more often presented with obvious colic signs requiring analgesics (grade 3) (*p* = 0.02), but without fever (*p* = 0.03) (Table 1). However, this was not clear from the cluster analysis, due to the few numbers of cases with grade 3 colic. By case definition, no palpable masses, thickened intestine or adhesions were recorded in the idiopathic cases. However, such rectal findings were present in 65% of horses confirmed with NSII lesions and showed close association with both confirmed and unconfirmed NSII cases in the cluster analysis. A normal rectal examination was significantly more common in the idiopathic case group (*p* < 0.001), with only one case diagnosed with NSII having no abnormal rectal findings. Trans-abdominal ultrasound was performed in 17 horses in the idiopathic peritonitis group, showing an increase in the amount of peritoneal fluid in all horses and in one case, also a mildly thickened small intestinal wall. No rectal ultrasound examination was performed in this group. In the NSII group, four horses had trans-abdominal ultrasound examinations, seven horses had rectal ultrasound examinations, and one horse had both types of examinations. On the trans-abdominal ultrasound, findings included increased amount of peritoneal fluid (*n* = 2), thickened small intestine (*n* = 3) and thickened colon wall (*n* = 1). All rectal ultrasound examinations showed a thickened colon wall, a localized mass involving or adjacent to a thickened colonic wall and/or suspected adhesion formation. Rectal ultrasounds were only performed if there had been abnormal rectal findings suggestive of a mass, thickened colon wall or adhesion formation. Remaining clinical data obtained at admission did not differ between groups (Suppl. Table 1c).

**Table 1:** Variables with significant differences between study groups as identified in nonparametric, univariable analysis. Odds ratio and 95% confidence interval calculated using univariable logistic regression. For rectal exam results, a univariable analysis of association for each type of rectal finding is included

*Variable*	*Idiopathic, n (%)*	*NSII, n (%)*	*P-value*		*OR*	*CI (95%)*	
**Season at presentation**^**a**^			0.001				
Spring/Summer	41 (38)	2 (10)	Reference variable				
Autumn	36 (34)	3 (15)		1.7			0.3–13.5
Winter	30 (28)	15 (75)		10.2			2.6–68.2
**Colic at admission**^**a**^			0.02				
No colic at admission	30 (28)	4 (20)	Reference variable				
Colic grade at admission							
* Grade 1*	67 (63)	12 (60)			1.50	0.48–5.67	
* Grade 2*	8 (7)	0 (0)			N/A	N/A	
* Grade 3*	2 (2)	4 (20)			17.50	2.62–161.78	
**Fever at admission (≥ 38.5 ℃)**^**a**^			0.03				
No fever at admission	53 (50)	4 (20)	Reference variable				
Fever at admission	52 (49)	16 (80)			0.3	0.1–0.8	
Missing	2	0					
**Rectal examination**^**a**^			< 0.001				
Normal rectal exam	54 (51)	1 (5)	Reference variable				
One or several rectal findings	53 (49)	19 (95)			19.4	3.8–354.0	
* Mass/adhesions*	0 (0)	13 (65)	Part of case definition				
* Impaction*	44 (41)	9 (45)			1.2	0.4–3.1	
* Gas distention*	7 (7)	4 (20)			3.6	0.9–13.3	
* Other abnormal finding*	4 (4)	1 (5)			1.4	0.0–9.8	
**WBC (median [IQR])**^**b**^	7.10 [5.54, 9.30] × 10^9^/L	3.50 [3.05, 5.01] × 10^9^/L	< 0.001		0.04^†^	0.28–0.64	
Missing	6	5					
**Neutrophils (median [IQR])**^**b**^	5.00 [3.60, 7.10] × 10^9^/L	2.40 [1.40, 3.19] × 10^9^/L	< 0.001		0.55^†^	0.38–0.75	
Missing	7	4					
**Elevated fibrinogen level**^**a**^	13 (16)	8 (53)	0.01		4.70	1.28–17.19	
Missing	23	5					
**Peritoneal total protein level (median [IQR])**^**b**^	40.00 [34.00, 50.00] g/L	50.00 [40.00, 60.00] g/L	0.01		1.38^‡^	1.10–1.78	
Missing	16	3					

^a ^Fisher exact test, ^b ^Kruskal-Wallis rank sum test, ^†^ for every increase of 1.0 × 10^9^/L, ^‡^ for every increase in 5.0 g/L

### Laboratory parameters and parasitic diagnostics

All blood samples included were obtained prior to surgical intervention. Elevated fibrinogen levels on first sampling occasion were significantly more common in the NSII group (Table 1, *p* = 0.01), but cluster analysis showed idiopathic cases with similar results (Suppl. Figure 1). Leukopenia, characterized by a neutropenia, was significantly more frequent in the NSII group compared to horses diagnosed with idiopathic peritonitis (*p* < 0.001) (Suppl. Figure 2a, 2b) and cluster analyses showed close association with final diagnosis. The mean protein level in the peritoneal fluid was higher in horses diagnosed with NSII as compared to the idiopathic case group (*p* = 0.01). However, as seen in the cluster analysis, there were horses in the idiopathic group with comparable levels. No other laboratory parameters were different between the two study groups (Suppl. Table 1d).

### Antimicrobial treatment regimes

All but one case in the idiopathic peritonitis group received antibiotic treatment. The horse that was not treated with antibiotics had peritoneal fluid with only a mildly elevated nucleated cell count (12.25 × 10^9^/L) and was discharged within five days from admission. The remaining 106 horses were treated with either intravenous penicillin G sodium (50%), penicillin G sodium and gentamicin (42%), penicillin G sodium and trimethoprim sulphonamide (6%) or various other combinations (2%). There was a difference in initial treatment regimen between the three hospitals, with the majority of horses at hospitals one and three treated with penicillin G sodium only (75% and 69%, respectively), whereas the broad-spectrum antibiotic combination of penicillin G sodium and gentamicin dominated at hospital two (78%). In the NSII group, four of cases were treated with penicillin G sodium only, all at hospital one. Six horses were initially treated with intravenous penicillin G sodium only, with the addition of either gentamicin (*n* = 5) or trimethoprim sulphonamide (*n* = 1) due to continuing fever (*n* = 4) or decision for surgery (*n* = 2). The remaining horses (*n* = 10) were treated with a combination of penicillin G sodium and gentamicin from admission.

### Progression of colic signs and fever during hospitalisation

In the idiopathic case group, the proportion of febrile horses (≥ 38.5 °C) decreased over the first 48 h of antimicrobial treatment from an initial 50%, to 18% at 24 h and further to 13% at 48 h. The proportion of idiopathic cases with grade 2 or 3 colic prior to medical treatment was low (9%), and decreased further over the first 24 and 48 h, to 6% and 4%, respectively. Overall, 16 cases (15%) diagnosed with idiopathic peritonitis had either fever or obvious signs of colic 48 h after initiation of antimicrobial treatment. In the NSII group, few cases had fever prior to antimicrobial treatment (25%), with six horses febrile at 24 h and three cases, at 48 h. Three horses showed colic at 24 h and another three cases at 48 h. At 48 h after antimicrobial treatment, six of the ten remaining horses (60%) exhibited either fever or obvious colic signs. The number of cases in this group diminished over the first 48 h as horses were either taken to surgery or euthanized. For specific data regarding colic signs and fever during the first 48 h of medical treatment for each individual NSII case, see Suppl. Table 2.

Of the unconfirmed cases, only a subset of horses showed obvious signs of colic or had documented fever over the first 48 h of antimicrobial treatment, with none of the horses surviving to discharge (*n* = 8) showing fever or colic at 48 h. Two of the three euthanized horses with unconfirmed NSII (no necropsy) were febrile 48 h after commencement of medical treatment.

Of the horses with palpable rectal masses that were not taken to surgery and subsequently did not survive to discharge (confirmed cases (*n* = 8) and unconfirmed cases (*n* = 3)), medical treatment was attempted over the first three to five days after admission in eight horses before a decision for euthanasia was made. In all eight cases, the horses continued to display intermittent fever and/or colic signs. Six of these cases subsequently had the diagnosis confirmed at necropsy. The remaining three cases with palpable rectal masses were euthanized the day after arrival due to the abnormal rectal findings and owner declination of surgery, without attempt at medical treatment. Two of these latter horses were confirmed with NSII at necropsy.

### Surgical findings

**Fig. 2:**
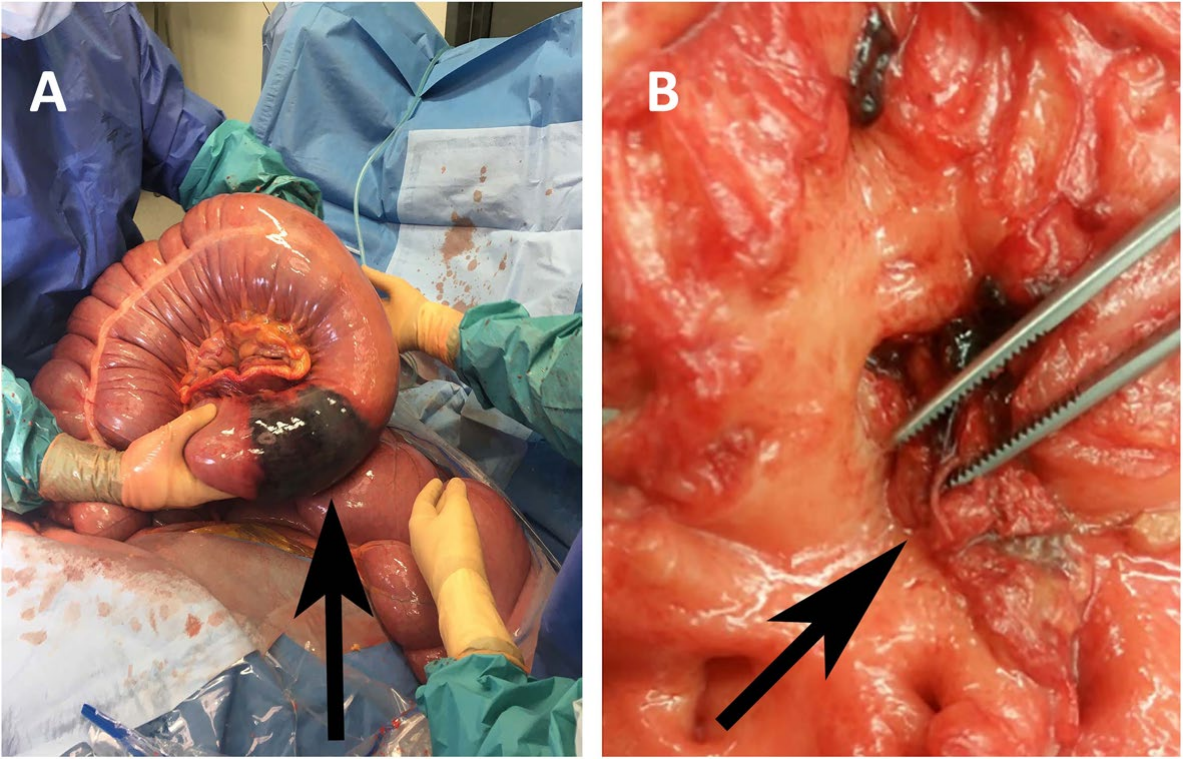
Panel **A**. An acute non-strangulating intestinal infarction in the left ventral colon. Panel **B**. Verminous arteritis of the cranial mesenteric artery with presence of *S. vulgaris* larvae

One horse in the idiopathic case group was subjected to surgery due to a right dorsal displacement of the ascending colon without vascular compromise. Another idiopathic case had an exploratory laparotomy performed, without abnormal findings. In the confirmed NSII group, all twelve cases where surgery was performed had marked changes involving a segmental portion of the pelvic flexure or the juncture of the dorsal colon and the pelvic flexure, with a clear demarcation between healthy and unhealthy bowel without signs of strangulation (Fig. 2, panel A). The compromised area of colon measured from five to 40 cm in length. In eight cases, thickening of the mesentery and/or enlargement of mesenteric lymph nodes were described. Adhesion formation between the pelvic flexure and the cecum and abdominal wall was found in three cases, of which one had a focal perforating lesion. Thrombosis formation in a ventral mesenteric vessel was observed in one horse.

### Outcome

All cases in the idiopathic peritonitis group were discharged after varying lengths of medical treatment, or medical treatment and surgical correction of a colon displacement in one case, with a survival to discharge rate of 100%. Most horses were discharged within 6–15 days of admission (70%). Twenty-seven horses (25%) were treated with antibiotics after discharge at home for a duration of two to eight days, although in 52% of these cases, treatment length at home was unknown.

**Fig. 3:**
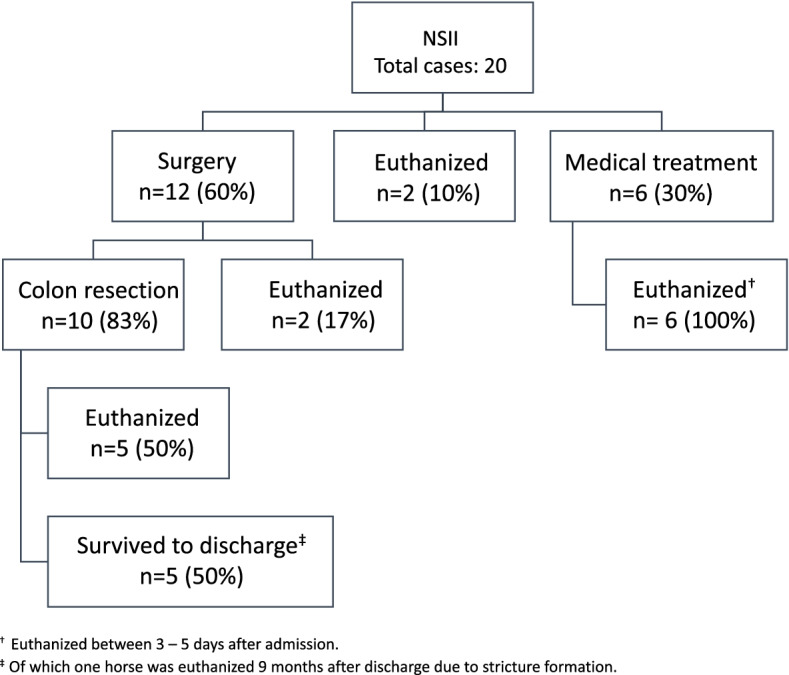
Outcome in horses with confirmed non-strangulating intestinal infarction (NSII)

In the NSII group, twelve horses (60%) had surgery performed between one to seven days after admission, with resection of the affected colon segment in ten cases. One horse was euthanized at surgery due to a ruptured bowel and another due to owner declination of colon resection. Five of the ten cases where colon resection had been performed survived to discharge, giving a short-time survival rate of 50% after colon resection. These cases had surgery performed at admission (*n* = 2), three (*n* = 1), four (*n* = 1) and seven days (*n* = 1) after presentation. One case that survived to discharge was subsequently euthanized nine months after surgery, due to stricture formation at the anastomosis site. Another five cases did not survive to discharge and were euthanized between 4 to 25 days after colon resection due to persistent colic signs or persisting septic peritonitis. Two horses that did not survive to discharge in addition developed wound infections, which, however, were not the reason for euthanasia. Eight cases, where surgery was declined by the owner based on the guarded prognosis, were euthanized one to five days from admission. A summary of the outcome in the NSII group is shown in Fig. 3. Horses with rectal findings suggestive of NSII had a survival rate to discharge of 38%.

### Pathology

#### Necropsy

In the NSII group, 9/20 (45%) of the horses had the diagnosis confirmed at necropsy, with the remainder confirmed after pathological examination of the surgically resected portion of intestine. In all cases, as defined by the inclusion criteria, the intestinal lesions were localized in or near the pelvic flexure. In most necropsied cases (7/9), a well-defined segmental area of transmural necrosis with varying degrees of eosinophilic inflammation of the pelvic flexure was demonstrated, in one case resulting in a perforating intestinal lesion. In one horse, the necropsy was performed after resection, where the resected intestine had not been sent for histopathological assessment. However, necrosis and ulcerations were observed at the anastomosis site and the caudal and cranial mesenteric roots showed severe lesions with thrombosis and multiple eosinophilic granulomas compatible with injuries secondary to *S vulgaris* larval migration. The segmental lesion in the final horse was described as a 40 cm long segmental haemorrhagic infarction with multiple ulcerations. The length of the affected portion of colon, when noted, was between 5 and 40 cm. Endarteritis of the cranial or caudal mesenteric roots and/or major mesenteric branches was found in all of horses, with thrombus formation recorded in seven cases. In six cases, *S. vulgaris* larvae were found within the arterial and/or intestinal lesions (Fig. 2, panel B). Reactive hyperplasia and haemorrhage of the mesenteric lymph nodes or eosinophilic lymphadenitis was observed in four of necropsied cases. In four cases, fibrinous to fibrous adhesions were found. In two of these horses, there was severe adhesion formation between the colon ascendens, the abdominal wall and colon descendens, involving the uterus in one case. An acute, subacute, purulent or fibrinopurulent peritonitis was recorded in all but one case, in which the necropsy was performed five days after resection of the affected portion of intestine.

One horse that underwent colon resection, with histopathological examination of the resected tissue, was subsequently sent for necropsy nine months later, after euthanasia, due to repeated colon impactions. A stricture formation was demonstrated at the anastomosis site, but there were no macroscopic lesions in the cranial or caudal mesenteric arteries or its branches. Histological evaluation of the mesenteric arteries was not performed.

#### Resected intestine

The remaining eleven cases in the NSII group, all underwent exploratory laparotomy with findings as described in the previous section. Colon resection was performed in all cases (one after euthanasia to confirm diagnosis) and histopathological evaluation of the resected intestine showed severe transmural lesions, with variable inflammatory, fibrinosuppurative to necrotic changes, in most cases with substantial eosinophilic inflammation. In nine cases, vascular lesions with thrombus formation were recorded. One of these cases was identified during surgery with thrombosis in a ventral mesenteric artery and in four cases, parasites consistent with *S. vulgaris* larvae were found.

## Discussion

It has previously been concluded that, in countries where *S. vulgaris* is considered endemic, all horses presenting with septic peritonitis without traumatic origin should be subjected to surgery [4]. In Sweden, however, as was shown in the present study, the vast majority of septic peritonitis cases are classified as idiopathic and respond to medical treatment with an excellent prognosis for survival [3]. The challenge with a possible increase in the number of peritonitis cases secondary to NSII lies in early detection, in order not to subject horses to surgery unnecessarily, to make an accurate prognosis, and to avoid suffering in cases where medical treatment is most likely hopeless, and where surgery or euthanasia instead would be the only feasible options. Although the number of confirmed cases of NSII in the present study was low, the data suggest that horses with septic peritonitis presenting during the winter months and where there is a palpable thickening of the colon and/or unidentified mass are very likely to have a NSII of the pelvic flexure secondary to *S. vulgaris* migration. A low blood neutrophil count, elevated fibrinogen levels and a high peritoneal protein level with fever or colic signs beyond 48 h of antimicrobial treatment may be further indications that surgery is indicated, but the substantial overlap in clinical and laboratory findings between the two case groups is likely to impede the use of these parameters in individual cases.

In accordance with a recent study by Odelros et al. (2019), idiopathic peritonitis cases were common and dominated at all three hospitals, with cases of confirmed NSII associated with vascular lesions caused by *S. vulgaris* larval migration less frequently diagnosed [3]. Similarly, a recent study in the UK found NSII to be a rare occurrence, although these cases were deduced to be of a different aetiology to *S. vulgaris* larval migration [7]. In Denmark, NSII was diagnosed in a minority of referred colic cases, with an incidence of 2% [4]. However, in the latter study, the authors noted an apparent increase in incidence since the implementation of a more selective approach to anthelmintic treatment and during the course of the study, horses presenting with peritonitis were increasingly taken to surgery at diagnosis, as clinicians became aware of the risk of NSII in this group of horses. In a country such as Sweden, where idiopathic peritonitis is common, this, however, could result in many cases taken to surgery unnecessarily. Nonetheless, in the present study, clinicians progressively recognized that horses presenting with both peritonitis and a palpable mass adjacent to the pelvic flexure were very likely to suffer from NSII and a decision for euthanasia in these horses, when the owners declined surgery, was often taken early during hospitalization, within five days of admission.

In the present study, there was a clear discrepancy in month of presentation, with idiopathic cases admitted uniformly throughout the year, whereas cases with confirmed NSII or a palpable rectal mass suggestive of NSII primarily presented during the winter months. This difference could be due to the underlying aetiology, i.e. the prolonged migratory phase of *S. vulgaris* larvae over a period of 4–5 months from infection [13], and similar findings have been made in other studies [4, 8]. In contrast, NSII cases of other aetiologies presented evenly over the year [7].

Interestingly, there were no differences between idiopathic and NSII cases in the proportion of horses that had received anthelmintic treatment within six months of admittance, with 35% of NSII cases reported to have been treated with an anthelmintic drug within this time. In a recent study, a similar percentage of horses were found to be positive on a faecal *S. vulgaris* PCR test, despite regular deworming at least annually or within six months of testing [12]. Possible explanations for these findings include re-infection from infected pastures after treatment or a lower than expected effectiveness of anthelmintic drugs on the L5 stage [14, 15].

Septic peritonitis, regardless of underlying cause, is associated with a systemic inflammation reflected in elevated inflammatory markers in the blood [2–4, 6, 16]. In the present study, the majority of horses had severely elevated SAA levels, with no differences detected between groups, supporting the common feature of acute systemic inflammation in both idiopathic peritonitis and NSII and in agreement with previous studies [3, 4]. In contrast, elevated fibrinogen levels at first sampling was more common in NSII horses. Fibrinogen increases more slowly than SAA in response to systemic inflammation, and the differences in fibrinogen level could reflect the duration of the disease process, with NSII cases presenting at a later stage [17].

In earlier studies, horses with NSII caused by verminous arteritis were described to present with acute colic, followed by endotoxemia and shock [18–21], similar to the description in a more recent case-report, where rupture of the ileum had occurred as a result of ischemic infarcted injury secondary to verminous arteritis [22]. In the present study, two horses developed signs of acute severe circulatory compromise soon after admission, in one case secondary to rupture of the colon, but none of the remaining 18 cases showed such signs. This is in accordance with Pihl et al. (2018), where none of the 30 included NSII cases showed signs of shock at presentation and similar to the idiopathic case group in the present study [4]. However, the total white blood cell count and neutrophil count differed between groups, with significantly lower neutrophil levels in the NSII cases. Neutropenia is a common feature of endotoxemia suggesting that, although often not obvious from the clinical examination, endotoxemia was more often present in horses diagnosed with NSII [23]. Analysis of blood and peritoneal fluid lactate levels were unfortunately only available in a minority of cases and could therefore not be included in the present study, but would have been of interest, in particular considering the underlying pathogenesis of NSII, resulting in severe circulatory compromise to the intestinal wall.

Although not a consistent feature, there was a palpable mass consistent with the location of the infarcted lesion in the majority of confirmed NSII cases in the present study. All intestinal infarctions were, as dictated by inclusion criteria, located in the pelvic flexure, which is an area readily accessible during rectal palpation, and highlights the importance of always performing a rectal examination in horses presenting with peritonitis, regardless of colic signs. Similarly, Pihl et al. (2018) found that in one third of the cases there was a palpable mass consistent with the location of the infarcted area and overall, 69% of the cases had an ischemic infarction located in the pelvic flexure [4]. However, NSII in other areas of the gastrointestinal tract, such as the small intestine or the apex of the cecum, would not be reachable at rectal palpation. Considering a poor correlation between palpatory findings and pathology of the mesenteric root has been shown, careful palpation of the large intestine nonetheless appears more useful in the diagnosis of NSII [5]. Transabdominal ultrasound was not helpful in diagnosis, but rectal ultrasound was a useful diagnostic aid in cases where colon thickening and/or a palpable mass had been detected. Ultrasonography of the cranial mesenteric artery could perhaps also be considered, especially in cases where no palpable mass is present [24].

During the first 48 h after initiation of antimicrobial treatment, there was a successive reduction in the proportion of idiopathic cases with either fever or obvious colic signs, to 15% of the total case load. In contrast, in the NSII group, 60% of cases that had not already been euthanized or taken to surgery had either documented fever or colic 48 h after medical treatment. However, since necropsy or pathological assessment of intestinal tissue was required for confirmation of NSII, only horses that subsequently were taken to surgery or were euthanized could be included in the NSII group. Thus, a major weakness in the study is that horses with NSII lesions that responded to medical treatment could have been missed. When considering all horses in the present study with rectal or ultrasonographic findings suggestive of NSII, regardless of confirmation, the overall short term survival rate was 37.5%, which is considerably lower than that of the idiopathic peritonitis case group (100%).

Eight cases with rectal, in some cases including ultrasonographic findings suggestive of NSII, survived with medical treatment alone and could represent horses in the early stages of NSII with subsequent healing of the infarcted area, as has been suggested previously [4, 25]. Whether some of the horses in the idiopathic case group also had smaller intestinal infarctions not palpable rectally and responding to medical treatment remains unverified. Parasitic migration of *S. vulgaris* with translocation of bacteria across the intestinal wall has been suggested as a possible cause of peritonitis in idiopathic cases [1, 2, 26], based on the isolation of *Actinobacillus* spp. from verminous aneurisms in the cranial mesenteric artery [27], highlighting the complexity in case differentiation. Recently, analysing serum for a *S. vulgaris* ELISA test, a significantly higher score was found in horses diagnosed with peritonitis compared to controls, even though the majority of cases were termed idiopathic [12].

## Conclusion

The results of the study supports medical treatment and a cautious approach to surgical decision in cases of peritonitis in countries such as Sweden where idiopathic peritonitis cases predominate. However, until specific markers for diagnosing *S. vulgaris* larval migration become available, clinicians working in countries where the parasite is endemic, should still be aware that horses presenting with septic peritonitis, particularly during the winter months, could suffer from intestinal infarction secondary to larval migration. In order to identify potential surgical cases, horses with septic peritonitis should be carefully evaluated, with particular emphasis on findings of rectal palpation and initial response to antimicrobial treatment.

## Methods

The study was conducted as a retrospective clinical study using patient records from three Swedish equine referral hospitals during the years 2017–2020. All procedures and treatments were directed by the treating veterinarian, and not for the purpose of this study.

### Case selection

For inclusion of idiopathic peritonitis cases, all cases with a diagnosis of peritonitis made within 48 h of admission were reviewed. Overall inclusion criteria were a peritoneal fluid cell count > 10 × 10^9^/L in horses where no identifiable cause for the peritonitis was determined from the clinical examination or at rectal palpation. Horses with palpable masses, thickening of the intestinal wall or suspected thromboembolism on rectal examination (in some cases also including rectal ultrasound examination) were excluded.

Regarding the NSII group, only horses with a confirmed diagnosis at necropsy or during explorative laparotomy were included. Criteria included a well demarcated ischemic lesion in close proximity of the pelvic flexure, which is an area with extensive collateral circulation and therefore prone to ischemia caused by arterial thromboembolism in the intestine. Horses had no signs of volvulus, other strangulating intestinal lesions or incarceration. Signs of verminous arteritis of the cranial or caudal mesenteric roots and its branches, as well as the presence of *S. vulgaris* larvae, were recorded if present, but not regarded vital for inclusion, since such lesion are difficult to ascertain without necropsy.

### Medical records review

Information obtained from the medical records is summarised in Table 2. In brief, information included demographic data, immediate history and clinical parameters at admission. Laboratory parameters included blood analyses, peritoneal fluid analyses and parasitic diagnostics. Hospital data regarding antimicrobial treatment regimen, outcome (non-survivor/days until discharge), surgical, pathological and necropsy reports were also used. Colic signs were graded as follows: grade 0: no colic; grade 1: decreased appetite/dull demeanour; grade 2: obvious colic signs (restlessness and pawing at the ground, irritated kicking to the stomach, rolling or attempting to roll); grade 3: obvious colic signs as grade 2 and requiring analgesics. Only grades 0–2 were used for grading of colic signs at home and grades 0–3 during hospitalization.

**Table 2:** Summary of data obtained from the medical records

*Parameter*	*Description*
Demographic data	Age, sex, breed
Immediate medical history	Colic signs^†^, rectal temperature, medical treatment (NSAID^a^, antibiotics), anthelmintic treatment past six months
Admission data	Month of presentation, heart rate, respiratory rate, mucous membranes (colour, crt^b^), colic signs^‡^, rectal temperature, rectal examination, abdominal/rectal ultrasound examination
Laboratory data	Blood: WBC^c^, neutrophil count, PCV^d^, TP^e^, SAA^f^, fibrinogen Peritoneal fluid: WBC^c^, TP^e^, bacterial culture Fecal sample: FEC^g^, *S. vulgaris* (PCR/culture), *A. perfoliata*
Hospital data	Colic signs^‡^, rectal temperature, antimicrobial treatment regime, days of hospitalization, outcome (non-survivor/days until discharge), surgical-, pathology- and necropsy reports

^†^ Graded as 0: no colic signs, grade 1: dull demeanour/anorexia, grade 2: obvious colic signs; ^‡^ Graded as 0: no colic signs, grade 1: dull demeanour/anorexia, grade 2: obvious colic signs, grade 3: obvious colic signs requiring analgesics

^a^non-steroidal anti-inflammatory drug; ^b^capillary refill time; ^c^white blood cell count; ^d^packed cell volume; ^e^total protein; ^f^serum amyloid A; ^g^faecal egg count

### Laboratory analyses

Analytical method used was primarily hospital dependent, but also dependent on the time of day a case was admitted (i.e. working hours or out-of-hours). Blood analyses for WBC and differential were performed by either Advia 2021i (Siemens, Erlangen, Germany), Procyte Dx (IDEXX, Hoofddorp, Netherlands) or Exigo H400 (Boule, Spånga, Sweden). Analyses for SAA was performed either by Architect C4000 (Abbot Core Laboratory, Abbott Park, Illinois, U.S.A.), Konelab 30i (Thermo Fisher Scientific, Waltham, U.S.A.) or StableLab (Epona Biotech Ltd, Sligo, Ireland) and fibrinogen by either Architect C4000 (Abbot Core Laboratory, Abbott Park, Illinois, U.S.A.) or Konilab 30i (Thermo Fisher Scientific, Waltham, U.S.A.). Peritoneal analyses for WBC was performed by either Advia 2021i (Siemens, Erlangen, Germany), Procyte Dx (IDEXX, Hoffddorp, Netherlands), Exigo H400 (Boule, Spånga, Sweden), Hemocue® WBC system (Radiometer, Crawley, England) or by manual count using a Bürker chamber. Peritoneal total protein was analysed using a refractometer (AO veterinary refractometer, U.S.A.).

Although different haematological analysers were used, total white blood cell counts and neutrophil counts were deemed comparable, since the analysers reference values were of similar magnitude. In the case of IDEXX Procyte Dx, the dot plot had to be normal for results to be included. Haematocrit, however, was excluded from the analysis since the different haematological analysers used were observed to render non-comparable values between cases. For the total nucleated cell count in the peritoneal fluid, results were presented within a specific range (10—30 × 10^9^, 30—150 × 10^9^, and > 150 × 10^9^), to account for the different analysers varying measuring ranges. Fibrinogen was reported to be above or below normal reference range, since different analysers prevented direct comparison of levels.

Of the horses where a bacterial culture of the peritoneal fluid was performed, 61% of the samples were analysed using a blood culture enrichment technique. In 76% of the samples, both aerobic and anaerobic culture was performed, with the remaining assessed by aerobic culture only.

### Parasite diagnostics

Strongyle faecal egg counts (FECs) were carried out for each horse using a modified McMaster technique with a minimum detection limit of 50 EPG [28]. *S. vulgaris* diagnostic method used was hospital dependent by either larval cultures on 50 g faeces according to Bellaw and Nielsen [29] and examined under the microscope using morphological criteria [30] or *S. vulgaris* specific PCR [11, 31]. *Anoplocephala perfoliata* were examined on 30 g faeces using the modified flotation technique described by Berozoa et al. [32].

### Statistical analyses

Excel was used for entering data and for descriptive analysis. Statistical analysis to study characteristics and potential indicators of horses with NSII was performed in R Statistical Software [33]. A cluster analysis including clinic, diagnosis, outcomes, horse characteristics and clinical information available upon admittance was performed [34, 35]. Variables were clustered by distance correlation. In addition, a descriptive and univariable analysis comparing horses with confirmed NSII and idiopathic peritonitis was performed. Quantitative measures were analysed using Kruskal–Wallis Rank Sum Test (non-parametric method) and qualitative variables using Fisher exact test (due to small number of observations) [36]. Odds ratios and 95% were calculated using univariable logistic regression [37, 38]. Seasonal effects of admittance of NSII patients were explored using a generalised additive model with a cyclic spline and clinic included as a random effect [39]. Level of significance was set at *p* < 0.05.

## Supplementary Information

**Additional file 1. **Data set all horses.

**Additional file 2: Suppl. Figure 1.** Cluster analysis. **Suppl. Figure 2 a.** Boxplot of total white blood cell count. **Suppl. Figure 2 b.** Boxplot of total neutrophil count.

**Additional file 3: Suppl. Table 1a.** Comparison of demographic data between non-strangulating infarction cases (NSII) and idiopathic cases using, for quantitative measures, the Kruskal-Wallis Rank Sum Test (non-parametric method) and, for qualitative variables, the Fisher exact test (due to small number of observations).

**Additional file 4:** **Suppl. Table 1b.** Relevant medical information showing variables non-significant between non-strangulating intestinal infarction cases (NSII) and idiopathic cases, using the Fisher exact test.

**Additional file 5:** **Suppl. Table 1c.** Admission data showing variables non-significant between non-strangulating infarction cases (NSII) and idiopathic cases, using, for quantitative measures, the Kruskal-Wallis rank sum test, and for qualitative measures, the Fisher exact test.

**Additional file 6: Suppl. table 1d.** Laboratory data showing variables non-significant between non-strangulating intestinal infarction (NSII) cases and idiopathic cases, using, for quantitative measures, the Kruskal-Wallis rank sum test, and for qualitative measures, the Fisher exact test.

**Additional file 7: Suppl. Table 2.** Presence of fever (if ≥ 38.5℃, indicated in red) and colic signs (if yes, indicated in red) in all individual non-strangulating infarction (NSII) cases before initiation of antimicrobial treatment, and 24 hours and 48 hours post-treatment.

## Data Availability

Original data is reported in Suppl. File “Data set all horses”. Subject to third-party agreement (horse owner), individual patient records can be available from the corresponding author on reasonable request.

## Abbreviations

NSII: Non-strangulating intestinal infarction
NSAID: Non-steroidal anti-inflammatory drug
SAA: Serum amyloid A
FEC: Faecal egg count
Epg: Eggs per gram
Crt: Capillary refill time
WBC: White blood cell count
PCV: Packed cell volume
TP: Total protein
