# Supplementary material for: Clinical features and treatment response to differentiate idiopathic peritonitis from non-strangulating intestinal infarction of the pelvic flexure associated with Strongylus vulgaris infection in the horse
Source: BMC Vet Res. 2022 Apr 23;18:149. doi: 10.1186/s12917-022-03248-x (PMC9034621; doi:10.1186/s12917-022-03248-x)
Supplement: Supplementary file 2 — Additional file 2: Suppl. Figure 1. Cluster analysis. Suppl. Figure 2 a. Boxplot of total white blood cell count. Suppl. Figure 2 b. Boxplot of total neutrophil count. [file 12917_2022_3248_MOESM2_ESM.docx]

Suppl. Figure 1. Cluster analysis.
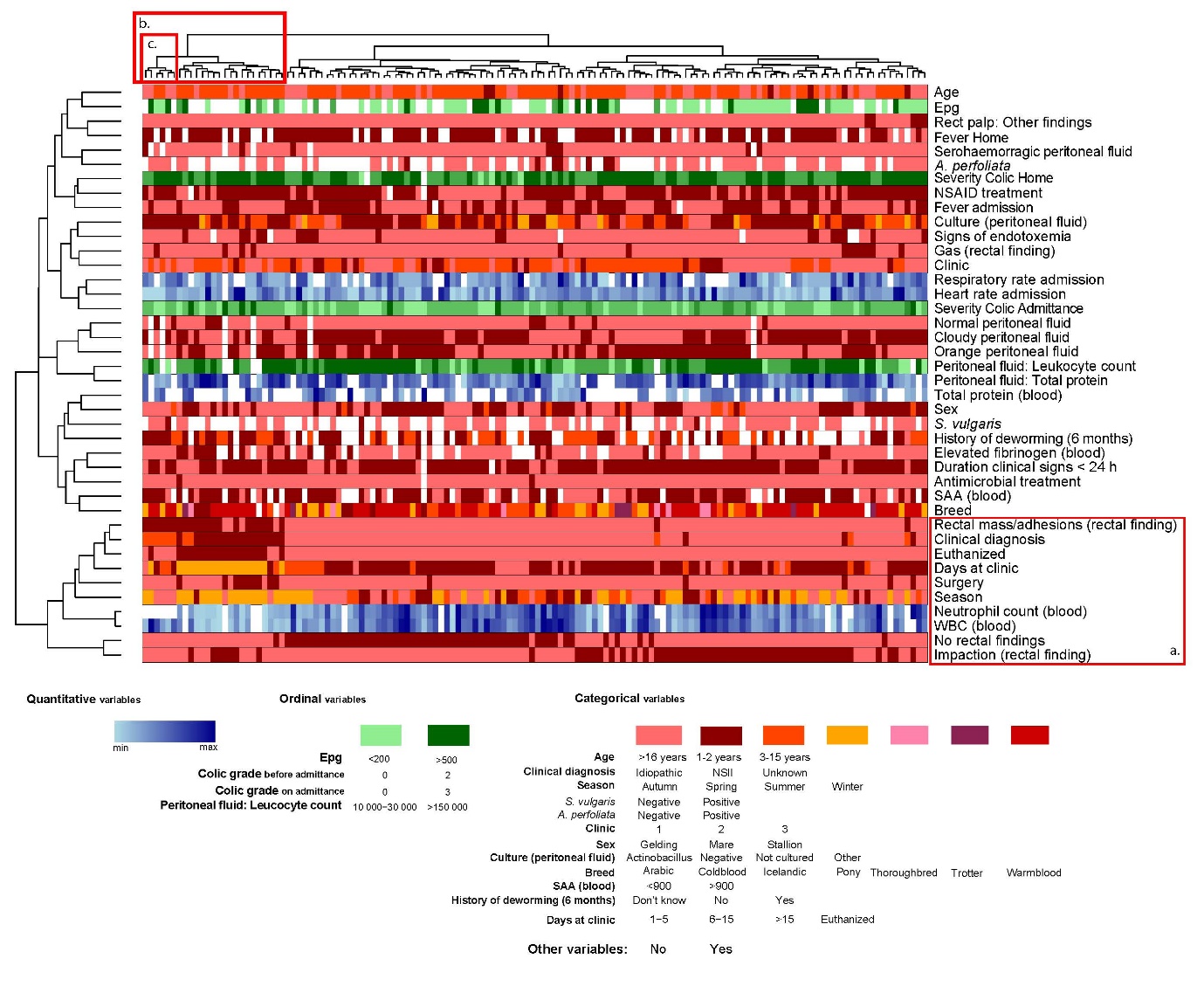


The top horizontal axis represents each individual case (clustered by similarity) and the case-based parameters (clustered by association) are presented on the left vertical axis. Results of the case-based value for each parameter are illustrated by a vertical line below each individual. Each cluster/subcluster is designated the letters a-c. Cluster a. Case-based parameters clustered by association. Cluster b. Individual cases clustered by similarity. Subcluster c. Subset of unconfirmed NSII cases.

Suppl. Figure 2 a. Boxplot of total white blood cell count.


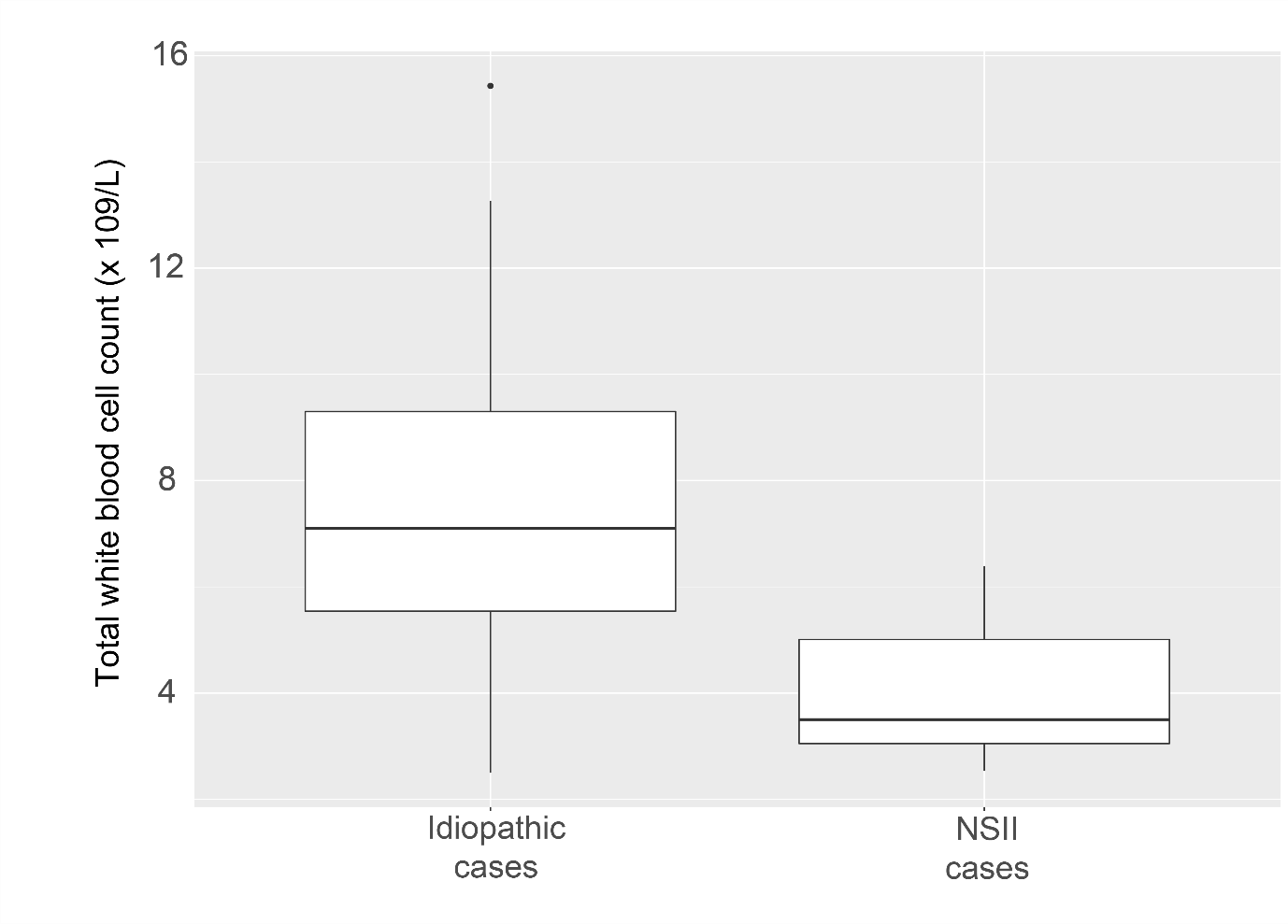


A boxplot illustrating the inter-quartile range (box) and minimum and maximum levels (vertical line) in total white blood cell count in idiopathic horses and horses with confirmed non-strangulating intestinal infarction (NSII). The bold line indicates the median. The dot indicates outlier.

Suppl. Figure 2 b. Boxplot of total neutrophil count.


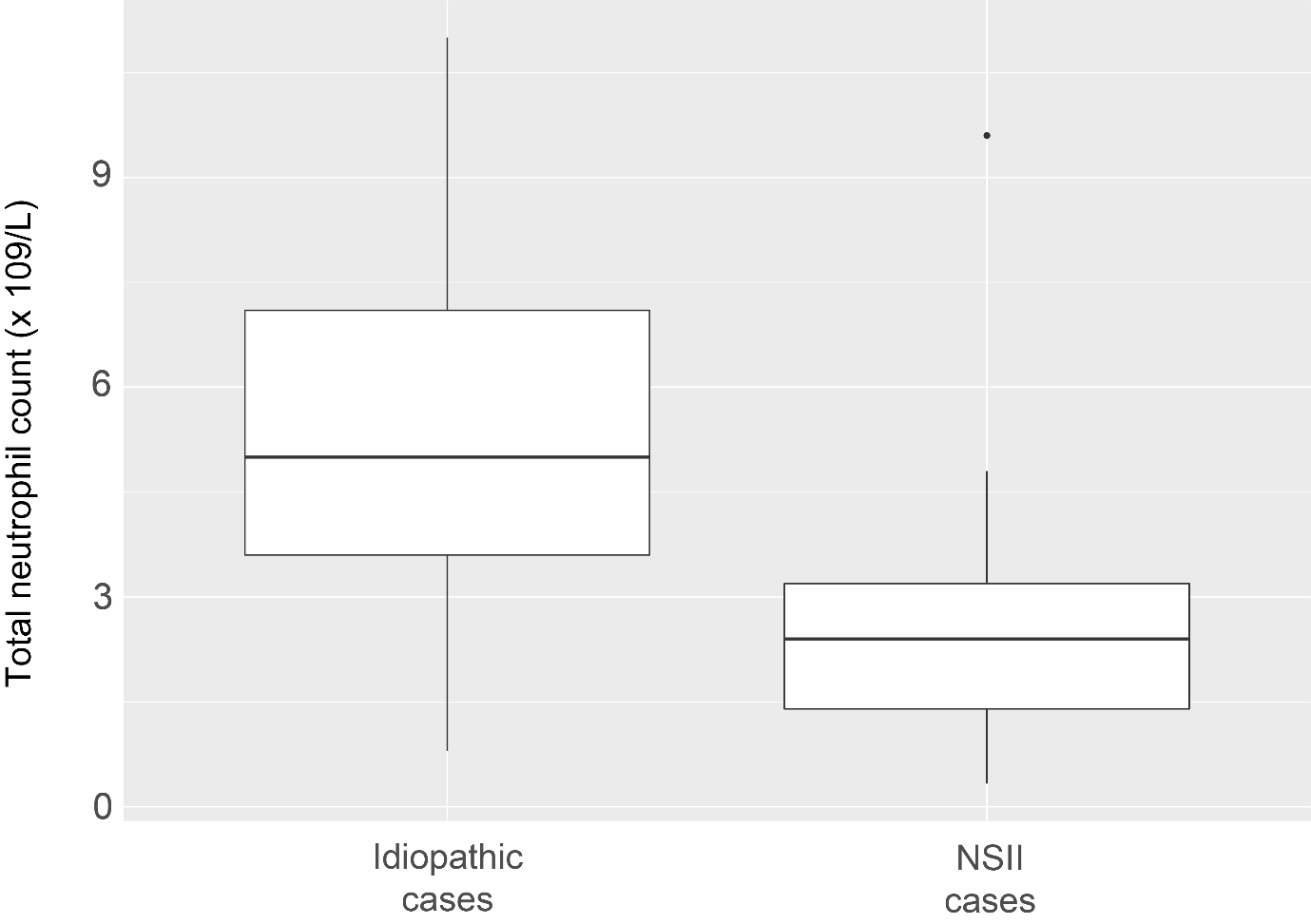


A boxplot illustrating the inter-quartile range (box) and minimum and maximum levels (vertical line) in total neutrophil count in idiopathic horses and horses with confirmed non-strangulating intestinal infarction (NSII). The bold line indicates the median. The dot indicates outlier.
