# Supplementary material for: Clinical features and treatment response to differentiate idiopathic peritonitis from non-strangulating intestinal infarction of the pelvic flexure associated with Strongylus vulgaris infection in the horse
Source: BMC Vet Res. 2022 Apr 23;18:149. doi: 10.1186/s12917-022-03248-x (PMC9034621; doi:10.1186/s12917-022-03248-x)
Supplement: Supplementary file 3 — Additional file 3: Suppl. Table 1a. Comparison of demographic data between non-strangulating infarction cases (NSII) and idiopathic cases using, for quantitative measures, the Kruskal-Wallis Rank Sum Test (non-parametric method) and, for qualitative variables, the Fisher exact test (due to small number of observations). [file 12917_2022_3248_MOESM3_ESM.docx]

| *Variable* | *Idiopathic, n (%)* | *NSII, n (%)* | *P-value* |
| --- | --- | --- | --- |
| Clinic^a^  One  Two  Three | 51 (48)  40 (37)  16 (15) | 10 (50)  8 (40)  2 (10) | 0.893 |
| Sex^a^ | | | 0.413 |
| Gelding | 52 (49) | 13 (65) |  |
| Mare | 47 (44) | 6 (30) |  |
| Stallion | 8 (7) | 1 (5) |  |
| Age^a^ | | | 0.838 |
| 1-2 years | 5 (5) | 1 (5) |  |
| 3-15 years | 68 (64) | 14 (70) |  |
| ≥ 16 years | 34 (32) | 5(25) |  |
| Breed^a^ | | | 0.055 |
| Warmblood | 53 (50) | 12 (67) |  |
| Coldblood | 5 (5) | 3 (17) |  |
| Icelandic Horse | 18 (17) | 1 (6) |  |
| Pony breed | 19 (18) | 1 (6) |  |
| Standardbred  Thoroughbred  Arabian | 6 (6)  5 (5)  0 (0) | 0 (0)  0 (0)  1 (6) |  |
| Missing | 1 | 2 |  |

Suppl. Table 1a. Comparison of demographic data between non-strangulating infarction cases (NSII) and idiopathic cases using, for quantitative measures, the Kruskal-Wallis Rank Sum Test (non-parametric method) and, for qualitative variables, the Fisher exact test (due to small number of observations).

^a^ Fisher exact test; ^b^ Kruskal-Wallis rank sum test
