# Supplementary material for: Clinical features and treatment response to differentiate idiopathic peritonitis from non-strangulating intestinal infarction of the pelvic flexure associated with Strongylus vulgaris infection in the horse
Source: BMC Vet Res. 2022 Apr 23;18:149. doi: 10.1186/s12917-022-03248-x (PMC9034621; doi:10.1186/s12917-022-03248-x)
Supplement: Supplementary file 4 — Additional file 4: Suppl. Table 1b. Relevant medical information showing variables non-significant between non-strangulating intestinal infarction cases (NSII) and idiopathic cases, using the Fisher exact test. [file 12917_2022_3248_MOESM4_ESM.docx]

| *Variable* | *Idiopathic, n (%)* | *NSII, n (%)* | *P-value* |
| --- | --- | --- | --- |
| Duration of clinical signs < 24 h | 87 (81) | 16 (80) | 0.753 |
| Missing | 2 | 0 |  |
| Colic at home | | | 0.216 |
| No colic | 4 (~~4~~) | 0 (0) |  |
| Grade 1^†^ | 46 (44) | 5 (25) |  |
| Grade 2^‡^ | 55 (52) | 15 (75) |  |
| Missing | 3 | 0 |  |
| Fever (≥ 38.5 °C) at home | 75 (90) | 12 (92) | 1 |
| Missing | 24 | 7 |  |
| Antibiotic treatment prior referral | 1 (1) | 1 (5) | 0.293 |
| Missing | 0 | 0 |  |
| NSAID treatment prior referral | 72 (69) | 16 (80) | 0.425 |
| Missing | 2 | 0 |  |
| Anthelmintic treatment within 6 months | | | 1 |
| No owner recollection | 3 (5) | 0 (0) |  |
| No | 35 (52) | 9 (53) |  |
| Yes | 29 (43) | 7 (44) |  |
| Missing | 40 | 4 |  |

Suppl. Table 1b. Relevant medical information showing variables non-significant between non-strangulating intestinal infarction cases (NSII) and idiopathic cases, using the Fisher exact test.

^†^ Dull demeanour/anorexia; ^‡^ Obvious colic signs; ^a^ Fisher exact test; ^b^ Kruskal-Wallis rank sum test
