# Supplementary material for: Clinical features and treatment response to differentiate idiopathic peritonitis from non-strangulating intestinal infarction of the pelvic flexure associated with Strongylus vulgaris infection in the horse
Source: BMC Vet Res. 2022 Apr 23;18:149. doi: 10.1186/s12917-022-03248-x (PMC9034621; doi:10.1186/s12917-022-03248-x)
Supplement: Supplementary file 5 — Additional file 5: Suppl. Table 1c. Admission data showing variables non-significant between non-strangulating infarction cases (NSII) and idiopathic cases, using, for quantitative measures, the Kruskal-Wallis rank sum test, and for qualitative measures, the Fisher exact test. [file 12917_2022_3248_MOESM5_ESM.docx]

| ***Variable*** | ***Idiopathic, n (%)*** | ***NSII, n (%)*** | ***P-value*** |
| --- | --- | --- | --- |
| **Hyperaemic mucous membranes^a^** | 13 (13) | 5 (28) | 0.141 |
| Missing | 3 | 2 |  |
| **Heart rate (median [IQR])^b^** | 46.00 [40.00, 52.00] | 44.00 [40.00, 54.00] | 0.787 |
| **Respiratory rate (median [IQR])^b^** | 16.00 [12.00, 20.00] | 14.00 [12.00, 20.00] | 0.393 |
| Missing | 20 | 4 |  |

Suppl. Table 1c. Admission data showing variables non-significant between non-strangulating infarction cases (NSII) and idiopathic cases, using, for quantitative measures, the Kruskal-Wallis rank sum test, and for qualitative measures, the Fisher exact test.

^a^ Fisher exact test; ^b^ Kruskal-Wallis rank sum test
