# Supplementary material for: Clinical features and treatment response to differentiate idiopathic peritonitis from non-strangulating intestinal infarction of the pelvic flexure associated with Strongylus vulgaris infection in the horse
Source: BMC Vet Res. 2022 Apr 23;18:149. doi: 10.1186/s12917-022-03248-x (PMC9034621; doi:10.1186/s12917-022-03248-x)
Supplement: Supplementary file 6 — Additional file 6: Suppl. table 1d. Laboratory data showing variables non-significant between non-strangulating intestinal infarction (NSII) cases and idiopathic cases, using, for quantitative measures, the Kruskal-Wallis rank sum test, and for qualitative measures, the Fisher exact test. [file 12917_2022_3248_MOESM6_ESM.docx]

| ***Variable*** | ***Idiopathic, n (%)*** | ***NSII, n (%)*** | ***P-value*** |
| --- | --- | --- | --- |
| **Peritoneal fluid leucocyte count^a^** | | | 0.32 |
| 10 – 30 x 10^9^/L | 13 (12) | 0 (0) |  |
| 30 – 150 x 10^9^/L | 42 (39) | 5 (33) |  |
| > 150 x 10^9^/L | 52 (49) | 10 (67) |  |
| Missing | 0 | 5 |  |
| **Gross appearance of the peritoneal fluid^a^** | | |  |
| Normal | 7 (7) | 3 (17) | 0.163 |
| Increased turbidity | 72 (69) | 8 (44) | 0.062 |
| Orange colour | 57 (54) | 12 (67) | 0.442 |
| Serohemorrhagic | 6 (6) | 1 (6) | 1 |
| Missing | 2 | 2 |  |
| **Peritoneal fluid culture^a^** | | | 0.376 |
| *Actinobacillus spp.* | 12 ( 11) | 0 (0) |  |
| Negative | 54 (51) | 11 (55) |  |
| Not cultured | 30 (28) | 8 (40) |  |
| Other bacterial growth | 11 (10) | 1 (5) |  |
| **Total protein level (serum) (median [IQR])^b^** | 66 [58.50, 69.50] | 60.50 [56.00, 65.50] | 0.269 |
| Missing | 52 | 12 |  |
| **Serum amyloid A (SAA)** |  |  |  |
| Level > 900 mg/L | 56 (66) | 13 (81) | 0.379 |
| **Fecal egg count (FEC)^a^** | | | 0.122 |
| <200 eggs per gram (epg) | 44 (69) | 7 (64) |  |
| 200-500 epg | 5 (9) | 3 (27) |  |
| > 500 epg | 16 (23) | 1 (9) |  |
| Missing | 42 | 9 |  |
| ***A. perfoliata*** **positive^a^** | 7 (12) | 0 (0) | 0.581 |
| Missing | 49 | 11 |  |
| ***S. vulgaris* positive^a^** | 3 (5) | 0 (0) | 1 |
| Missing | 50 | 11 |  |

Suppl. table 1d. Laboratory data showing variables non-significant between non-strangulating intestinal infarction (NSII) cases and idiopathic cases, using, for quantitative measures, the Kruskal-Wallis rank sum test, and for qualitative measures, the Fisher exact test.

^a^ Fisher exact test; ^b^ Kruskal-Wallis rank sum test
