# Supplementary material for: Clinical features and treatment response to differentiate idiopathic peritonitis from non-strangulating intestinal infarction of the pelvic flexure associated with Strongylus vulgaris infection in the horse
Source: BMC Vet Res. 2022 Apr 23;18:149. doi: 10.1186/s12917-022-03248-x (PMC9034621; doi:10.1186/s12917-022-03248-x)
Supplement: Supplementary file 7 — Additional file 7: Suppl. Table 2. Presence of fever (if ≥ 38.5℃, indicated in red) and colic signs (if yes, indicated in red) in all individual non-strangulating infarction (NSII) cases before initiation of antimicrobial treatment, and 24 hours and 48 hours post-treatment. [file 12917_2022_3248_MOESM7_ESM.docx]

| ***Horse ID*** | ***Before treatment*** | | ***At 24 hours*** | | ***At 48 hours*** | |
| --- | --- | --- | --- | --- | --- | --- |
|  | ***Fever*** | ***Colic***^†^ | ***Fever*** | ***Colic***^†^ | ***Fever*** | ***Colic***^†^ |
| 109 | 38.5 | No | 37.3 | Yes | Surgery |  |
| 110 | No fever | No | Missing | Yes | 37.2 | Missing |
| 111 | 37.3 | No | Surgery |  |  |  |
| 112 | 37.2 | No | 38.3 | Yes | 37.8 | No |
| 113 | 37.8 | No | Surgery |  |  |  |
| 114 | 37.8 | Yes | Surgery |  |  |  |
| 115 | 39.0 | Yes | Surgery |  |  |  |
| 116 | 37.9 | Yes | Surgery |  |  |  |
| 117 | 37.8 | No | 37.7 | No | 37.4 | No |
| 118 | 37.0 | Yes | Surgery |  |  |  |
| 119 | 38.1 | No | 38.5 | No | Euthanized |  |
| 120 | 37.4 | No | Euthanized |  |  |  |
| 121 | 37.6 | No | 39.0 | No | Euthanized |  |
| 122 | 37.7 | No | 37.6 | No | 37.4 | Yes |
| 123 | 38.5 | No | 37.6 | No | 38.8 | No |
| 124 | 37.7 | No | 38.9 | No | 38.2 | Yes |
| 125 | 37.6 | No | 39.2 | No | 39.5 | No |
| 126 | 37.7 | No | 39.7 | No | 39.3 | No |
| 127 | 39.1 | No | 38.3 | No | Missing | Yes |
| 128 | 39.3 | No | 38.5 | No | 38.3 | No |

Suppl. Table 2. Presence of fever (if ≥ 38.5℃, indicated in red) and colic signs (if yes, indicated in red) in all individual non-strangulating infarction (NSII) cases before initiation of antimicrobial treatment, and 24 hours and 48 hours post-treatment.

^†^ Colic defined as either grade 2: obvious colic signs or grade 3: obvious colic signs requiring analgesics
